# The Bermuda Triangle of paediatric brain cancers: epigenetics, developmental timing window and cell of origin

**DOI:** 10.1007/s10555-025-10284-0

**Published:** 2025-09-23

**Authors:** Afraah Cassim, Yolanda Colino-Sanguino, Sarah L. Fox, Laura Rodriguez de la Fuente, Hannah E. Hartley, Fatima Valdes-Mora

**Affiliations:** 1 Cancer Epigenetic Biology and Therapeutics Group, Children’s Cancer Institute, Sydney, NSW 2052 Australia; 2 School of Clinical Medicine, Faculty of Medicine & Health, University of New South Wales Sydney, Kensington, NSW 2052 Australia; 3 School of Biomedical Engineering, Faculty of Engineering and Information Technology, University of Technology Sydney, Ultimo, NSW 2007 Australia

**Keywords:** Paediatric brain cancer, Cell of origin, Epigenetics, Brain development, Diffuse high-grade gliomas, Ependymomas

## Abstract

Paediatric brain cancers are aggressive tumours that urgently need deeper understanding of their cellular and molecular vulnerabilities to facilitate the development of effective treatments. These tumours frequently arise from epigenetic alterations in specific immature cell states of the developing prenatal or neonatal brain. In this review, we propose a “three-event” model composed of an epigenetic event, developmental timing window and the cell of origin for tumour initiation in paediatric brain tumours. We focus on three types of paediatric gliomas: diffuse midline gliomas (DMG), diffuse hemispheric gliomas (DHG) and posterior fossa A ependymomas (PFA-EPN), which reflect our proposed three-event model. Additionally, we discuss the methods and models used to study these three events separately or simultaneously. Taken together, this review highlights the spatio-temporal vulnerable cell states during brain development and which molecular drivers hijack these cues to induce cell state stalling and tumour initiation. The next steps to expand our understanding of the order of events and their use in therapy are further discussed.

## Introduction

Unlike adult cancer, paediatric cancers are developmental diseases [1]. Particularly in the developing brain both prenatally and postnatally, mutations, usually in epigenetic factors, are key drivers of paediatric brain cancers [2]. In fact, the convergence of such genetic/epigenetic alterations, a specific developmental window, and a particular cell type context are the three prerequisites for vulnerability to tumour formation. We have called this phenomenon the “three-event model of paediatric brain cancer”. In addition to this model, some paediatric brain tumours also require secondary cooperative mutations to drive and maintain tumourigenicity [3]. In this review, we examine the specific alterations in the paediatric intracranial brain tumours diffuse midline gliomas (DMG), diffuse hemispheric gliomas (DHG) and posterior fossa group A ependymoma (PFA-EPN), which fit the three-event model. We will discuss how the processes of the developing brain are hijacked through the epigenetic aberrations that occur in these cancers to promote tumourigenesis. We will conclude how the understanding of these processes is paving the way towards better treatments.

### Overview of the developing brain and epigenetics

**Fig. 1:**
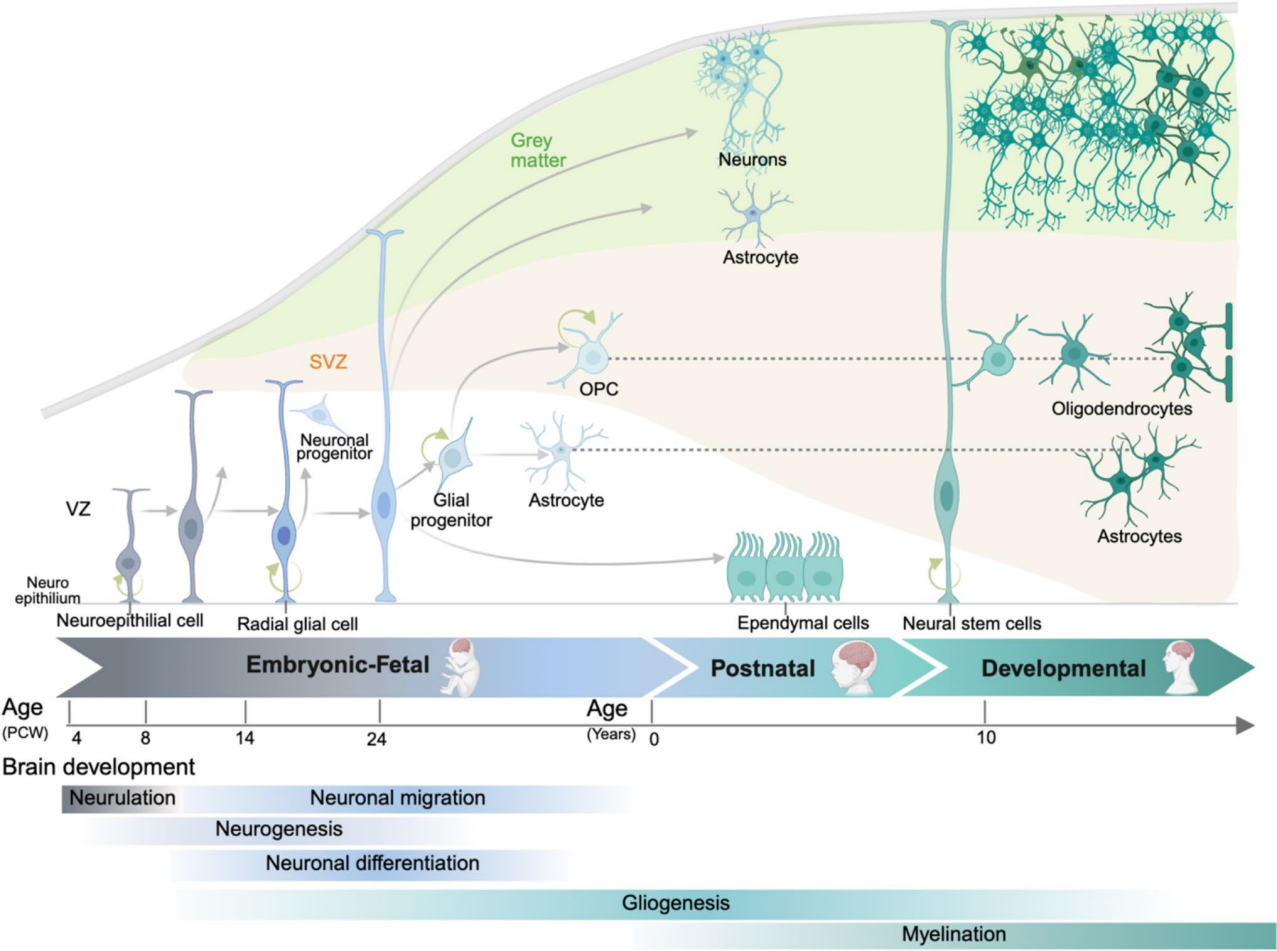
Representation of normal neurogenesis and gliogenesis from the embryonic to the adult phases of human brain development. Neuroepithelial cells transform into radial glial cells, to produce neuronal progenitors and later undergo a glial fate switch to become intermedial glial progenitor cells. These cells can produce astrocytes or OPCs later in the embryonic phase. The postnatal phase involves the production of ependymal cells from the radial glial cells and further production of neurons, oligodendrocytes and astrocytes. The color of each cell matches the developmental phase (embryonic, postnatal or towards adult development). VZ ventricular zone, SVZ subventricular zone, OPC oligodendrocyte precursor cell, PCW postconceptional weeks

**Fig. 2:**
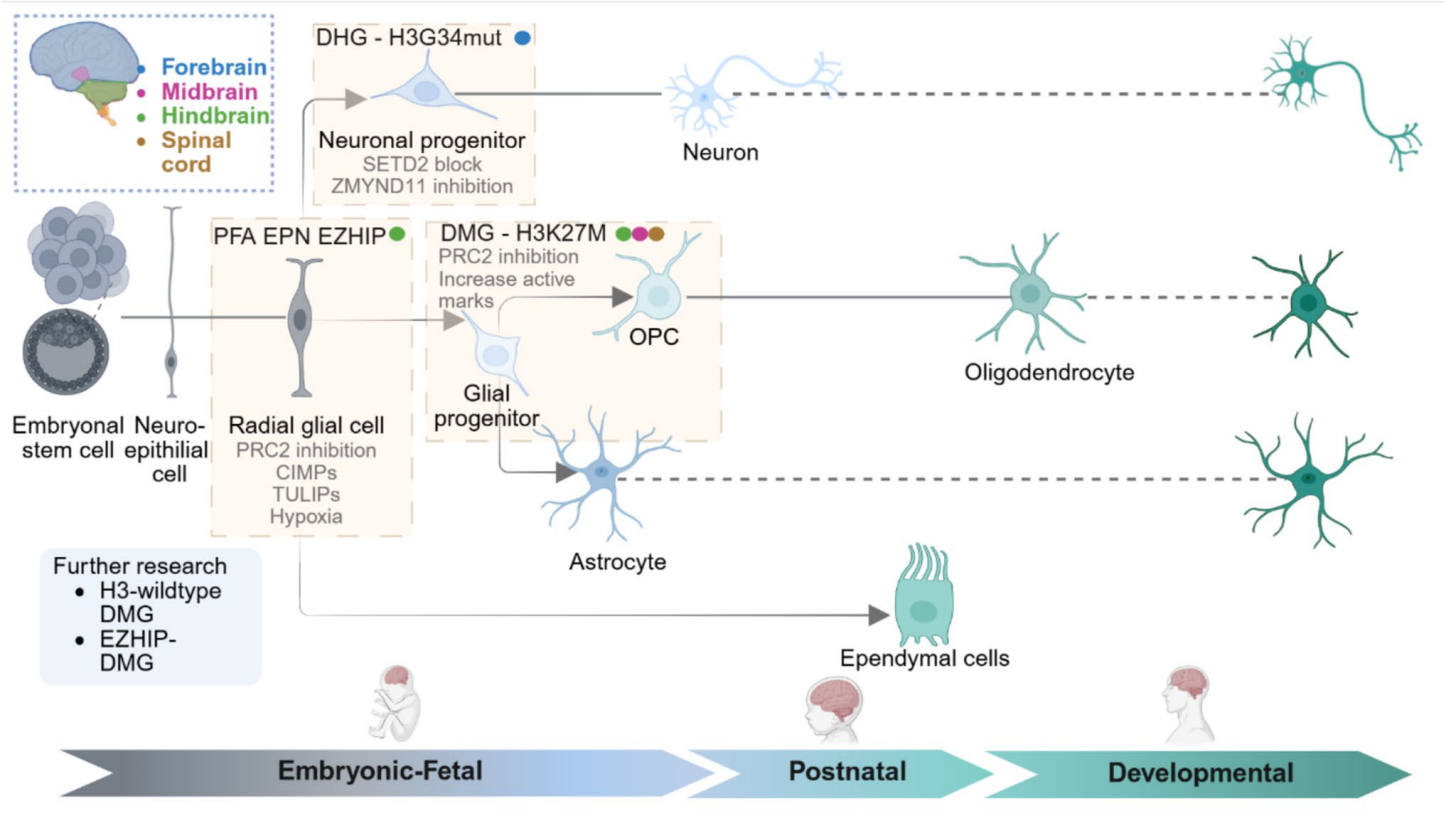
Putative cell of origin and epigenetic drivers in paediatric brain tumours. This schematic illustrates the proposed cells of origin and predominant epigenetic drivers (listed in grey) for three major paediatric brain tumour subtypes: posterior fossa group A ependymoma (PFA-EPN), diffuse hemispheric glioma (DHG) and diffuse midline glioma (DMG). Proposed cell of origin in PFA-EPN is radial glial cells, where overexpression of EZHIP together with a hypoxic environment leads to inhibition of the PRC2 complex, resulting in a global reduction of H3K27me3. This is accompanied by a CpG island methylator phenotype (CIMP) marked by widespread promoter hypermethylation and silencing of differentiation-related genes. Recent 3D genome analyses show that EZHIP-driven remodelling induces long-range B-compartment loops (“TULIPs”) that reinforce repression at key developmental loci. DHG, frequently marked by H3.3G34R/V mutations, likely originates from intermediate neuronal progenitors in the developing forebrain. The G34 mutations disrupt the function of SETD2, impairing H3K36me3 deposition and altering chromatin readers and transcriptional repressor ZMYND11 causing loss of transcriptional repression at key neuronal progenitor genes. Finally, DMG (H3K27M expressing) putative cell of origin is intermediate glial progenitors within the oligodendrocyte lineage. The H3K27M mutation dominantly inhibits PRC2, causing a widespread but incomplete loss of H3K27me3 and a compensatory gain of H3K27ac. This leads to a distorted epigenetic landscape that favours proliferation and prevents normal lineage progression. Coloured dots indicate the neuroanatomical region associated with each tumour type: forebrain (blue), midbrain (pink), hindbrain (green) and spinal cord (gold). Dotted lines represent prolonged development. Future work is needed to resolve the developmental origins of other DMG subtypes, including EZHIP-DMG and H3-wildtype DMG

Brain development is a highly complex and dynamic process that occurs in several stages, broadly encompassing neurogenesis, cell migration, differentiation, maturation, synaptogenesis, pruning, and myelination. The human brain starts developing prenatally and spans through adulthood; however, the most rapid growth occurs during the early years [4] (Fig. 1). Coincidentally, paediatric brain cancer is the second most common cancer in children, and its aetiology is mostly attributed to alterations during brain development [1]. Here, we present an overview of the neurodevelopmental events and their associated epigenetic mechanisms that occur prenatally and during childhood to provide the context of the developmental origins of these cancers (Fig. 2).

In humans, during the third and fourth gestational weeks, brain development commences with the formation of the neural tube where neural stem cells (NSCs) proliferate and differentiate, to give rise to the three major structures of the brain: the forebrain (containing the cerebral hemispheres), midbrain, and hindbrain (containing the brainstem and cerebellum) [4]. There are two main cell lineages in the nervous system: neurons and glial cells, which consist of hundreds of neuronal and glial subtypes (oligodendrocytes, astrocytes and ependymal cells) with a common precursor, NSCs. Neurogenesis and gliogenesis are highly intricate processes in which NSCs develop into distinct types of brain cells; neurons and glial cells, respectively. These processes are time and brain region-dependent [5] (Fig. 1). For example, during embryonic neurogenesis the NSCs called neuroepithelial progenitors (NEPs) form the inner lining of the neural tube and the hypoxic conditions of the neural tube promote the progression of NEPs towards a multipotent NSC called radial glial cells (RGCs). As embryogenesis continues, RGCs differentiate into neuronal progenitor cells which ultimately originate neurons that migrate to posterior layers (Fig. 1) and will specialise into specific neuron types, depending on the region they locate and their final function. Later in embryonic development and during the perinatal stages, gliogenesis takes place from RGCs, which give rise to astrocytes and, postnatally, to oligodendrocytes and ependymal cells [6] (Fig. 1).

The human brain is highly heterogeneous in the context of cell types and specialised functions, and during the different stages throughout brain development, cell types require a precise and controlled regulation of gene expression that is mediated by both external factors and intrinsic factors, including hypoxic environments or the glial or neuronal switches (Fig. 1) [6]. Epigenetic enzymes and mechanisms (Box 1) play a critical role in the spatial and temporal gene expression regulation in response to these extracellular and intrinsic cues to induce proliferation, differentiation and fate commitment of the cell types from the central nervous system (CNS) during human brain development [7]. Both global epigenetic changes and discrete chromatin changes at master regulator genes for differentiation have been described to be intimately linked to cell fate and are specific to each cell type, anatomic location and developmental stage. Epigenetic changes during brain development have been comprehensively reviewed elsewhere [7, 8]. Here, we will focus on a few examples of major epigenetic events that occur in brain development and that have been shown to be altered in paediatric brain cancer: DNA methylation and histone acetylation and methylation (Box 1).

Global DNA methylation is highly dynamic during neurogenesis, with waves of gains and losses during different stages and dependent on the cell type, and thus it remains to be fully resolved [9]. For example, RGCs undergo consecutive waves of DNA methylation loss. Thefirst demethylation occurs at genes involved in the induction of neuronal progenitors, later another wave of demethylation occurs at the promoters of glial genes involved in astrogliogenesis. On the other hand, glial cells will also gain *de novo* methylation at neuron-specific genes to lock in the glial fate [10]. As a clear example of the critical role of DNA methylation in neurogenesis, studies have demonstrated that differential DNA methylation patterns, but not gene expression profiles, can identify the different cell lineages of the brain [10], and in the context of brain cancer, DNA methylation is used to subclassify these tumours through the so-called “DKFZ Classifier”, as it directly  reflects the cell of origin [11].

Chromatin bivalency is characterised by the co-occurrence of the repressive mark H3K27me3 and active mark H3K4me3 and is a critical process during embryonic development to regulate rapid responses to external cues and to maintain pluripotency [12] (Box 1) This bivalency is commonly found in NSCs and oligodendrocyte precursors [13].

Histone acetylation at the lysine 27 is a mark of active enhancers (Box 1), and studies in neurogenesis have shown that this mark is highly dynamic and becomes progressively restricted as neural differentiation occurs [14]. The CBP and p300 histone acetyltransferases (HATs) that acetylate H3 and H4 at different lysines, regulate neurogenesis by controlling the differentiation of RGCs into astrocytes/oligodendrocytes presumably through their recruitment by master regulators of cell fate, like Neurogenin [15, 16]. This suggests that HATs and their interaction with different transcription factors are key for the regulation of broad gene expression programs that establish cell fate through enhancer/gene activation.

Histone 3 lysine 27 trimethylation (H3K27me3) is a critical chromatin repressive mark for brain development, and, like DNA methylation, it is highly dynamic throughout neurogenesis [8]. For example, the timing of EZH2 loss in RGCs during corticogenesis affects the fate of these cells. When EZH2 is deleted before neurogenesis begins, it accelerates neural lineage progression by reducing H3K27me3 levels, which in turn increases overall gene expression [17]. Conversely, deleting EZH2 during neurogenesis extends the neurogenic phase and delays the transition to astrogliogenesis [18].

Experiments knocking down epigenetic factors like DNMT1, EZH2 and CBP/P300 have shown contrasting phenotypes of either cellular stalling or acceleration of cell differentiation (reviewed [8]), which can be explained by the different cell type context and is in line with the waves of epigenetic regulation during brain development. Therefore, it is critical to understand the cell of origin and its anatomical context to explain how epigenetic alterations that occur in paediatric brain cancers are driving tumourigenesis.

**Table:** 

**Box 1. Epigenetics and Cancer Epigenetics ** Epigenetics involves molecular processes that modify the DNA without altering the original nucleotide sequence regulating gene function and ultimately cellular activity [19]. Epigenetics is essential for the normal functioning of a cell and when it is dysregulated leads to disease, including cancer. There are many epigenetic mechanisms that regulate chromatin structure; however, we will focus on the main epigenetic processes that are affected in paediatric brain cancers: DNA methylation and histone modifications by methylation or acetylation. Epigenetic modifications are dynamically regulated by three principal epigenetic factors: “*writers*” which deposit epigenetic marks, “*erasers*” which remove them and “*readers*” which interpret these modifications to direct downstream transcription.	
**1. DNA Methylation** DNA methylation involves the covalent addition of a methyl group to the cytosine bases of CpG dinucleotides. Global hypomethylation of intergenic regions contributes to genomic instability and oncogene activation. Focal hypermethylation at promoter regions of classical tumor suppressor genes (*MGMT, RB1, CDKN2A*) and DNA repair genes (*BRCA1*), lead to their transcriptional silencing [20]. ***Writers*** **:** DNA methyltransferases (DNMT1, DNMT3A, DNMT3B**)** catalyse the formation of 5-methylcytosine (5mC) marks [21], which anchor methyl-CpG-binding domain (MBD) transcriptional repressor proteins. ***Erasers*** **:** The removal of methyl groups by oxidation of 5mC is mediated by Ten-eleven translocation (TET) enzymes TET1/2/3, facilitating transcriptional reactivation [22]. TET mutations, or mutation of metabolic enzymes including isocitrate dehydrogenase (IDH) inhibit TET catalytic activity driven oncogenesis [23].	
**2. Histone Modifications in Histone H3** Histone H3 is subject to dynamic post-translational modifications (PTMs) on its N-terminal tails, namely methylation, acetylation and ubiquitination. These modifications regulate chromatin architecture to define ‘active’ euchromatin versus ‘repressive’ heterochromatin transcriptional states.	
**2a. Histone Methylation** Histone methylation occurs on lysine (K) or arginine (R) residues and can be activating or repressive, depending on the site and methylation degree (mono-, di-, tri-methylation). • **Repressive marks**: H3K9me3, H3K27me3, define a repressive heterochromatin state. • **Activating marks**: H3K4me1, H3K4me3 and H3K36me3 define open euchromatin states promoting active transcription. These modifications reside at specific regulatory regions, H3K4me1 marks enhancers; H3K4me3 marks promoters and H3K36me3 marks gene bodies of highly transcribed genes, constituting transcriptional elongation. • **Bivalent chromatin**: Bivalent chromatin domains, made up of simultaneous asymmetric H3K4me3 and H3K27me3 marks, dictate a poised gene state for repression or activation of cell-lineage specific transcription programs [24]. Specifically, KMT2C and PRC2/1 establish bivalent domains [25, 26], providing a mechanism by which dysregulation of respective epigenetic regulators may promote oncogenesis. Indeed, disruption of finely balanced chromatin bivalence is linked to tumor dedifferentiation, therapy resistance and immunomodulation [26].	
**2a.1. Key mediators** ***Writers:*** Polycomb group (PcG) proteins are essential chromatin-modifying factors that mediate transcriptional silencing of key developmental and cell identity genes through the formation of multiprotein polycomb repressive complexes (PRC), PRC2 and PRC1. Of particular importance is the Polycomb Repressive Complex 2 (PRC2), a multiprotein complex composed of the catalytic subunit EZH2, along with SUZ12 and EED. PRC2 has a histone methyltransferase activity primarily through EZH2 which tri-methylates H3K27 (H3K27me3). PRC2 is central to developmental gene regulation, silencing lineage-specific transcriptional programs and maintaining cellular identity [20]. Another key epigenetic writer, SETD2, is a histone methyltransferase responsible for depositing H3K36me3, playing a critical role in maintaining chromatin integrity and regulating transcriptional elongation, DNA damage repair, and RNA splicing [27]. Loss-of-function mutations or deletions in *SETD2* (*KMT3A*) are recurrent in various cancers leading to global epigenetic dysregulation and oncogenesis by impairing DNA mismatch repair and altering the expression of tumor suppressor genes [27, 28]. ***Erasers*** *:* Removal of methylation from histones is controlled by histone demethylases (KDMs). KDM1A (LSD1) removes methyl groups from H3K4 and H3K9, whilst KDM6A/B (UTX/JMJD3) specifically demethylate H3K27me3. Loss or mutation of KDMs can lead to persistent gene silencing, epigenetic plasticity, and dedifferentiation; hallmarks of many cancer types. ***Readers:*** ZMYND11 functions as a chromatin reader, specifically recognising and binding to histone H3.3 trimethylated at Lys-36 (H3.3K36me3). Additionally, ZMYND11 regulates RNA polymerase to promote transcriptional expression patterns crucial for tumor growth [29].	
**2b. Histone Acetylation in Histone H3** Histone acetylation is another major epigenetic modification with distinct implications for chromatin accessibility and transcriptional activity. Acetylation of lysine residues on histones H3 neutralises their positive charge, weakening the interaction between histones and DNA. Resulting euchromatin structure permits transcription factor binding and gene activation. • **Key marks:** H3K27ac marks active enhancers and super enhancers which are strongly associated with oncogene activation. H3K9ac is enriched at active promoters.	
**2b.1. Key mediators** ***Writers*** **:** The writers of histone acetylation include several histone acetyltransferase (HAT) families: GNAT, MYST, and the CBP/p300 complex. The latter plays a prominent role in enhancer activation through H3K27ac. ***Erasers***: histone deacetylases (HDACs), remove activating acetylation marks particularly H3K27ac to restore a repressive heterochromatin state. The balance between HAT and HDAC activity is crucial for maintaining gene expression programs. ***Readers***: Acetylated lysines are recognised by Bromodomain (BRD) containing and Extra Terminal domain (BET) protein families, BRD2-4 and BRDT, which recruit transcriptional co-activators and machinery to acetylated histones [30], particularly to oncogenic super enhancer sites.	

## Diffuse midline gliomas, *H3 K27-altered* (DMG H3K27a)

Diffuse midline gliomas, H3 K27-altered (DMG H3K27a) are a highly aggressive type of gliomas within the family of paediatric-type diffuse high-grade gliomas and are defined by their anatomic location within midline structures of the central nervous system: pons, thalamus and spinal cord; their histological presentation, a diffusely infiltrative growth pattern with glial lineage features; and their molecular features, global loss of the epigenetic mark H3K27me3 (Box 1) [31, 32]. Although classified as a single entity in the 2021 World Health Organization (WHO) Classification, DMG H3K27a is still an heterogeneous subtype. This variability arises primarily from differences in the prerequisites of the three-event model (epigenetic alteration, developmental window and cell of origin) and the spectrum of secondary driver mutations [33], which together contribute to molecular and cellular diversity that underlies distinct clinical features, including variation in median age at diagnosis and patient prognosis [3, 34–36]. In the next section, we will describe DMG H3K27a based on epigenetic alterations, the developmental window where these alterations occur, and their cell of origin.

### Epigenetic alterations in DMG H3K27a

DMG H3K27a is an epigenetically driven cancer with a common hallmark of a global loss of the epigenetic repressive mark H3K27me3 and it is further subclassified into four subtypes [35, 37]: (1) diffuse midline glioma, H3.3 K27 mutant: results from a somatic mutation that substitutes lysine 27 with methionine at the *H3F3A* gene that encodes the H3.3 variant (DMG H3.3K27M, majority of cases)) [38]; (2) diffuse midline glioma H3.1 or H3.2 K27-mutant: a somatic mutation that substitutes lysine 27 with methionine at the *HIST1H3B* and *HIST2H3C* genes, respectively (DMG H3.1/2K27M) [3]; (3) diffuse midline glioma H3 wild type with EZHIP overexpression: results from an aberrant expression of the enhancer of zeste inhibitory protein (EZHIP) (DMG EZHIP, [34, 35, 39]) and (4) diffuse midline glioma *EGFR* mutant: results from both insertion/deletion within exon 20 or missense mutations at the alanine 2989 replaced by threonine valine (A289T or p-A289V) at the *EGFR* gene (DMG-EGFR) [40–42]. The latter subtype has been discovered very recently, and the EGFR mutations mostly co-occur either with H3 K27 mutations or EZHIP overexpression [35], but some cases that are negative for H3K27M and EZHIP have been also found [42].

The loss of H3K27me3 is directly caused by either H3K27M or EZHIP. Mechanistically, H3K27M and EZHIP, which contains a domain that mimics H3K27M [43, 44], both act as a dominant negative inhibitor of the Polycomb Repressive Complex 2 (PRC2), [45–47]. How H3K27M and EZHIP inhibit PRC2 has been the cause of intense investigation and it has been concluded that they act through the disruption of the allosteric activation and catalytic cycling of the PRC2 complex, exerting a dominant-negative effect that extends beyond the local chromatin environment [46, 48–54]. Although PRC2 remains recruited to high-affinity nucleation sites such as CpG islands, contact with H3K27M-containing nucleosomes traps the PRC2 complex in a non-productive state, preventing the read–write mechanism necessary for H3K27me3 spreading [53, 54]. A key component of this inhibition is the blockade of EZH2 automethylation [55], a modification required for full enzymatic activity [56]. H3K27M disrupts automethylation even after transient interactions with PRC2, leaving the complex in a hypoactive state long after dissociating from mutant nucleosomes or peptides [55, 57]. Even though the majority of studies have been focused on the most abundant H3.3K27M alteration or have been agnostic to the different isoform context [46, 48–55], peptide assays showed that the K27M mutant histones bind to EZH2’s active site in both variants [46]. However, H3.1K27M has a higher impact on PRC2 activity compared to H3.3K27M, potentially due to differences in genomic distribution [45]. In line with this, human NSCs overexpressing either H3.1K27M or H3.3K27M showed different sensitivity toward EZH2 inhibition, where H3.3K27M seem to be more reliant on remaining PRC2 activity [58].

Similarly to H3K27M, EZHIP interacts with the PRC2 complex, particularly the chromatin bound and allosterically stimulated PRC2 where EZHIP inhibits H3K27me3 spreading [54]; however, more research is needed to understand EZHIP’s mode of action as most studies that investigate PRC2 inhibition in DMGs have exclusively focused on H3K27M.

The alteration of H3K27me3 levels has been associated with developmental stalling through two mechanisms, loss of H3K27me3 at bivalent promoters of cycling and pluripotent or stem cell-like genes [58–60] and retention, or even gain, of H3K27me3 at strong PRC2 targets at promoters of neurodevelopmental genes, such as the Hox cluster [52, 61]. H3K27me3 retention has also been detected in a small subset of PRC2 positive poised enhancers [51, 61].

DMG H3K27a tumours exhibit additional widespread epigenomic changes. However, it remains unclear whether these alterations arise directly from the presence of H3K27M or EZHIP, and/or are the consequence of cellular stalling induced by PRC2 inhibition. For example, the total levels of H3K27 acetylation are dramatically elevated [46, 52, 61, 62] across the genome, including at repetitive elements which results in an increase in their expression [61, 62]. In contrast, H3K27ac decreases at enhancers of NSC differentiation genes that are downregulated when H3K27M is overexpressed in human hindbrain neural stem cells[61]. Interestingly, chromatin accessibility is also reduced at those enhancers, including NOTCH1. These results contrast with other reports where ATAC-seq in isogenic DMG cell lines showed that H3K27M cells acquire new open chromatin regions [62, 63], particularly at enhancers and super enhancers of genes controlling neurogenesis and NOTCH signalling [63]​. A recent paper that used a different patient-derived DMG cell line showed that removal of H3K27M had transcriptional and chromatin changes that did not occur in the EZH2 KO, suggesting that H3K27M alters chromatin accessibility independently to PRC2 [64]. However, other studies have shown that H3K27M does not mimic a complete inhibition of PRC2 [51, 58, 61], suggesting that the changes in transcription and chromatin accessibility may differ between EZH2 KO and H3K27M KO. Therefore, the effect of H3K27M in chromatin accessibility remains unclear.

H3K27M has also been shown to exert a direct effect on H3K4me3 deposition independent to PRC2 inhibition [65]. Mutant H3K27M-containing nucleosomes aberrantly recruit the H3K4 methyltransferase MLL1, leading to widespread redistribution and accumulation of H3K4me3 at distal regions. This enrichment promotes higher levels of bivalent chromatin at the remaining H3K27me3 sites and contributes to maintaining an undifferentiated state. This is the first study to attribute alterations in a histone modification, other than H3K27me3, to a direct effect of H3K27M, rather than to secondary consequences of disrupted H3K27me3 deposition.

DNA methylation profiling can stratify H3K27 altered DMGs from other paediatric high-grade glioma [11], and can also distinguish between H3.1K27M and H3.3K27M tumour samples [66, 67] and EZHIP overexpressing and EGRF positive subtypes [35], suggesting a different cell of origin. Bender et al. reported that in K27M-mutant gliomas, upregulated genes frequently exhibit both DNA hypomethylation and H3K27me3 loss (~ 30%), whereas only a minority of downregulated genes show DNA hypermethylation (~ 15%), suggesting that not all DNA methylation changes are driven by H3K27me3 loss or lead to transcriptional effects. A study that investigated DNA methylation entropy across individual H3K27 altered DMG cells found higher level of DNA methylation variability at genes involved in pluripotency and developmental cell identity compared to normal foetal brain, potentially enabling cells to sample diverse transcriptional programs and differentiation states [68]. While these findings highlight distinct DNA methylation patterns, it remains unclear to what extent DNA methylation changes arise from different cellular origins versus being directly driven by H3K27M or other subtype-defining alterations.

Studies of DMG’s 3D chromatin architecture have revealed a distinct spatial organisation compared to normal cell lines and other brain tumours such as glioblastoma [69] and ependymoma [70]. However, no studies to date have examined 3D chromatin conformation in H3K27M loss-of-function or overexpression models to demonstrate if H3K27M drives those changes or are a result of a different cellular origin.

In summary, H3K27M or EZHIP causes loss of PRC2 function and unleashes an epigenetic deregulation cascade that results in gene expression changes of neurodevelopmental regulators and cell lineage stalling.

### Developmental window and the cell of origin of DMG H3K27a

Histologically, DMG H3K27a present with undifferentiated glial cells which led to the speculation that these gliomas arose during the glial differentiation in the developing brain; however, determining the specific developmental window and if these windows are different for each subtype has been subject to increasing investigation over the last 14 years [1, 47, 71, 72]. This has been challenging because H3K27M/EZHIP-induced transformation may require not only the presence of the H3K27M mutation/EZHIP expression in a narrow developmental window but also a precise susceptible progenitor cell.

The first question that has been under debate for years is whether H3K27M/EZHIP alterations occur pre- or post- natally, and the second question is which critical cell type is  susceptible to oncogenic transformation.

To answer the first question, sophisticated *in vitro* and *in vivo* models of H3K27M tumours have been created (Box 2, Table 1); however, conflicting results are observed where both prenatal or postnatal windows for tumour initiation are suggested. In pursuit of a postnatal window of origin, two attempts to induce tumours reflective of H3K27M-driven DMG were unsuccessful when use of a retroviral Replication-Competent ASLV long terminal repeat (LTR) with a Splice acceptor (RCAS) system to induce H3.3K27M alone in neural progenitor cells in the postnatal mouse brain did not produce tumours in one study. However, when combined expression of PDGFB with H3.3K27M, high-grade gliomas were successfully produced [73]. Interestingly, the injection of transduced neural precursor cells with H3.3K27M plus PDGFRA and sh-p53, into the pons of immunocompromised mice only produced low-grade tumours [47]. These results suggest that the induction of H3K27M may occur prenatally, or it needs to be induced in other cell types rather than NSCs or neural progenitor cells.

**Table 1:** Summary of the key models and methods to study the three-event model in epigenetically driven paediatric brain tumours. NA: not available, further research needed

Paediatric brain cancer family	Epigenetically driven tumour type	Epigenetically driven tumour subtype	Driver epigenetic event	Key methods used to study the epigenome	Development window and anatomic location	Key models to study developmental window	Cell of origin	Key methods to study the cell of origin
Paediatric-type diffuse high-grade gliomas	Diffuse midline glioma, H3 K27-altered	Diffuse midline glioma, H3.3 K27-mutant	H3.3 K27M oncohistone mutation	ChIP-seq, HiC [69, 101]	Window: E12.5–13.5 P0–4 (mouse) Location: brainstem midline	*In vitro*: by differentiation of stem cells [47, 58, 61, 78] *In vivo*: GEMM [59, 74] and patient xenografts [71, 106]	Neural progenitor, dorsal PAX3^+^/BMP-dependent progenitors	scRNA-seq [1, 72, 76, 77] H3K27ac ChIP-seq [78, 85]
		Diffuse midline glioma, H3.1 or H3.2 K27-mutant	H3.1/H3.2 K27M oncohistone mutation	ChIP-seq, ATAC-seq, HiC, [69]	Window: NA Location: brainstem, midline	NA	NKX6-1^+^/SHH-dependent brainstem OPC	scRNA-seq [77] H3K27ac ChIP-seq [78]
		Diffuse midline glioma, H3-wildtype with *EZHIP* overexpression	EZHIP expression	IHC [34] DNA methylation [35]	Window: NA Location: once defined as thalamic tumours	NA	NA	NA
		Diffuse midline glioma, *EGFR*	H3K27M or EZHIP expression	IHC, DNA methylation [35, 107]	NA	NA	NA	NA
	Diffuse hemispheric glioma, H3 G34-mutant	NA	H3.3 G34-RV oncohistone mutation,	ChIP-seq [108]	Window: second trimester, or shortly after birth (human) Location: Fore brain	*In vitro*: [85, 89, 90, 93] *In vivo* Xenografts: [106]	Interneuron progenitor cells	scRNA-seq [90, 93]
Ependymal tumours	Posterior fossa group A (PFA) ependymoma	NA	EZHIP expression (95%) or H3K27M (5%)	DNA methylation [109], ChIP-seq [43, 98, 101]	Window: E10–E16 (mouse) Location: fourth ventricle and cerebellum	*In vivo*: patient xenograft models [110, 111]	Radial glial cell	scRNA-seq [76, 101, 103]

The presence of secondary mutations seems to be a prerequisite for tumour formation when creating mouse models of DMG. By harnessing the development period of when gliogenesis occurs (P0–P4), Larson and colleagues generated diffuse midline gliomas by inducing a genetically engineered triple mutation (H3.3K27M mutation, *PDGFRA* mutant and *Tp53* loss) in Nestin positive neural stem/progenitors of neonatal mice (P0–P4) [59]. Another study induced the expression of H3.3K27M mutation, *PDGFRA/PDGFRB* and loss of *Tp53* in either Nestin + or Olig2 + progenitors during the neonatal window in immunocompetent mice. While all models generated tumours, only the Nestin + H3.3K27M progenitors notably reduced tumour latency, while in the Olig2 + progenitors no differences were observed between H3WT and H3.3K27M tumours, owing to the conclusion that Nestin + cells (NSCs) are more vulnerable to H3K27M-driven postnatal transformation [74].

In consideration of a prenatal window for transformation, studies where H3.3K27M expression was induced in murine embryonic stem cells, it was lethal at very early stages of development and, similarly, the conditional expression of H3.3K27M in NPCs—whether alone or combined with Tp53 deletion—both prenatally and postnatally, failed to induce tumours. However, when H3.3K27M and co-drivers were induced at specific stages of development in NPCs and at particular locations, such as the hindbrain (day E12.5) and forebrain (E13.5) regions, through *in utero* electroporation, tumourigenesis was successfully induced with 100% penetrance [75]. These findings suggest that H3.3K27M and secondary mutations are induced prenatally and are dependent on the specific development window to lead to tumour transformation postnatally.

In summary, certain methods to induce H3K27M-DMG in both prenatal and postnatal windows have been successful in recapitulating DMGs *in vivo*. However, the differences in models and co-driver mutations have been large confounding factors, leading to an ineffective comparison and the main question of pre/postnatal window still needing further investigation.

**Fig. 3:**
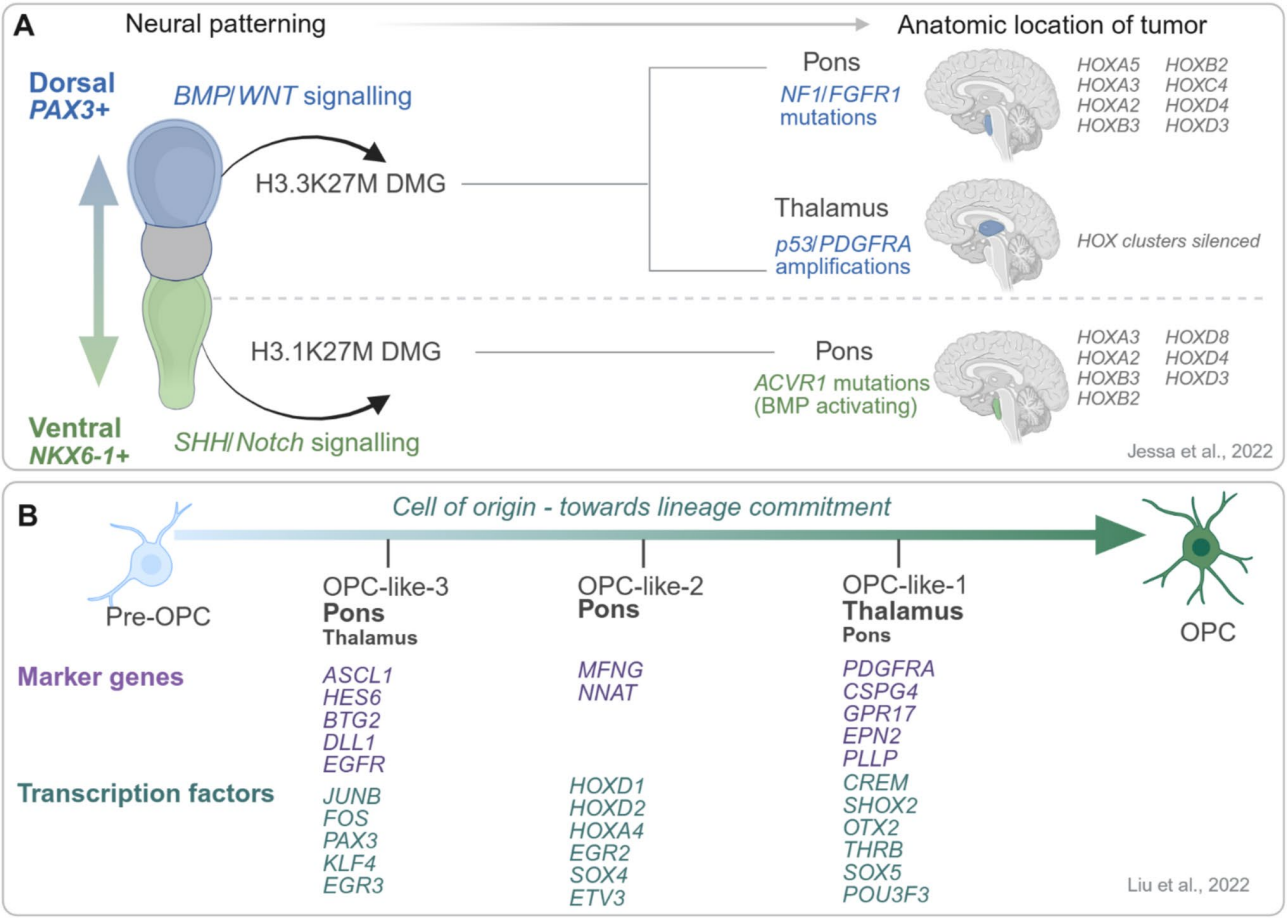
Overview of recent studies to understand the early development and cell of origin in H3K27M-DMG tumours. **A**. Early development: Neural tube morphogen patterning with dorsal and ventral expression segregating H3.3 and H3.1K27M tumour development. Anatomical location of tumour coincides with co-driver mutations and *Hox* gene expression, with *Hox* gene silencing in thalamic tumours contributing to regional specification of the tumour [77]. **B**. Cell of origin: Overview of tumour heterogeneity between the pre-OPC and OPC cell states [76]. Majority of more differentiated OPC-like-1 cells are found in thalamic tumours, while less differentiated OPC-like-2,3 are found predominantly in the pons. Marker genes for OPC-like-1,2,3 are shown in purple, and specific transcription factors are shown in dark green

With regard to answering the second question of the cell of origin for oncogenic transformation in DMG, there has again been contrasting results, with some studies stating a neural progenitor cell [47, 58, 59, 61, 71, 74], astrocyte origin [76] or oligodendrocyte origin [72, 76–78]. Advances on high-throughput single cell omics and the increase of samples available from DMG patients from biopsies or autopsies have allowed the precise characterisation of the cell of origin for H3.1K27M and H3.3K27M DMGs from a developmentally stalled early oligodendrocyte precursor cell state [72, 76–78]. These studies showed that H3K27M tumours presented with varying maturity of stem-like oligodendroglial precursor cells across all clinico-anatomical groups of age and tumour location [76]. Further insights from a single-cell multiomic study, which included H3K27ac and H3K27me3 ChIP-seq data, suggest that H3.1K27M and H3.3K27M DMGs arise from OPCs derived from distinct spatial domains of the neural tube (Fig. 3A), and that H3K27M-DMG tumours were highly correlated towards the pre-OPC, OPC-2,3 less differentiated states [76] (Fig. 3B).

The H3.1K27M DMGs are enriched for ventral sonic hedgehog (SHH) signalling progenitors expressing *NKX6-1* and possess a super-enhancer regulating *NKX6-1* expression. This reflects the first wave of oligodendrocyte fate specification of ventral NKX6 +, with the potential of differentiating to ependymal cells and astrocytes as seen in single cell RNA-sequencing of this subtype. In contrast, pontine H3.3K27Ms arise from dorsal progenitors that are governed by PAX3 + and BMP/WNT signalling [77], and super-enhancer analysis demonstrates that one of the core transcription factors in H3.3K27M DMGs is PAX3 [1]. This reflects the second wave of oligodendrocyte fate patterning in the dorsal PAX3 + region. The chromatin architecture around the HOX cluster is also indicative of the location and developmental time where the genetic alteration occurred. HOX gene activation is distinct between H3.1K27M and pontine H3.3K27M tumours, as well as thalamic H3.3K27M, where *HOX* genes are silenced (Fig. 3A). In fact, gene expression patterns suggest that H3.3K27M thalamic gliomas arise exclusively from p2 domain of the embryonic diencephalon that give rise to the thalamus, rather than the classic dorsal or ventral domains that pattern the brainstem and spinal cord.

The pattern of co-occurring mutations in H3K27M-DMGs also provides critical clues about the developmental context and lineage of the cell of origin. In H3.1K27M DMGs, which arise predominantly in the pons, the frequent co-occurrence of *ACVR1* mutations suggests a ventral OPC origin influenced by SHH signalling, as ACVR1-mediated BMP pathway activation is necessary to support proliferation and self-renewal in this ventral lineage [77]. By contrast, H3.3K27M pontine gliomas typically harbour mutations in *Tp53* and amplifications of *PDGFRA*, a receptor tyrosine kinase highly expressed in dorsal OPCs (Fig. 3A) [76, 77]. H3.3K27M thalamic gliomas often exhibit *FGFR1* gain-of-function mutations rather than *PDGFRA* alterations. Interestingly *FGFR1* mutations are associated with various low-grade neuroepithelial tumours, suggesting the progenitors originating H3.3K27M thalamic gliomas have lower gliogenic potential. In this regard, the cell of origin in other H3K27a DMG tumours remains elusive, with no current models of EZHIP-DMG for disease modelling in this context posing further research questions.

Together, these findings underscore that the oncogenic potential of the H3K27M mutation is highly context-dependent, requiring the convergence of a permissive developmental time window, a susceptible progenitor identity and cooperating secondary mutations. As discussed previously, a specific prenatal or postnatal window has not yet been confirmed. Given the successful transformation into DMG in Nestin + cells both *in vitro* and *in vivo*, there is still the unanswered question if the cells are in fact most epigenetically vulnerable at the Nestin + neural precursor state (cell of mutation) [58, 59, 74], but may proceed to and stall at the OPC state, which reflects the recent transcriptomic data [1, 76, 77].

## Diffuse hemispheric glioma, H3 G34-mutant (DHG-H3G34mut)

Diffuse hemispheric gliomas H3 G34-mutant** (**DHG-H3G34mut) have recently been reclassified as their own distinct tumour type within the paediatric-type diffuse high-grade gliomas family by WHO [32] and comprises 15% of all HGGs [79]. This type of grade 4 tumour primarily arises in the cerebral hemispheres and has a median survival of less than 2 years after diagnosis and a median age at diagnosis of 15.8 years [32, 80, 81]. DHG H3G34mut is defined by a somatic missense mutation in the *H3F3A *gene leading to glycine-to-arginine or valine substitutions at position 34 (G34R/V) [3, 32]. Other alterations that are frequently found in DHG are mutations of *ATRX* and *TP53*,* PDGFRA*,* EGFR*,* CDK4*,* CDK6* genes or *CCND2* amplification, *CDKN2A/B* homozygous deletion and *MGMT* promoter methylation [80–82]. Paediatric patients with DHG-H3G34mut tumours have longer survival than H3K27a or IDH and H3 wild-type DMGs [79]. Within DHG tumours, patients carrying G34V, rather than R mutations and *PDGFRA* or *EGFR* amplification, and lacking *MGMT* promoter methylation have been associated with the poorest prognosis [82].

### Epigenetic alterations in DHG H3G34mut

Unlike DMG-H3K27M, H3G34 mutation is exclusive to the gene that encodes for histone variant H3.3, and the different location of the mutation within the gene and its substitution result in very different global epigenomic changes. Biochemical studies showed that H3.3G34R mutation blocks the access to the neighboring lysine 36 by the methyltransferase SETD2 inhibiting the trimethylation of H3K36, a mark of active transcription (Box 1), and this inhibition, unlike in DMG H3K27a, does not translate to global changes in H3K36me3, but rather at specific loci [46]. Another direct consequence of G34R is the preferential binding and inhibition of KDM4 demethylation activity which usually removes H3K9 and H3K36 methylation, and results in gain of the repressive mark H3K9me3 and H3K36 methylation in KDM4 bound sites but not necessarily at the chromatin sites where mutant H3.3 occurs [83]. The reduction of H3K36me3 has been shown to be associated with an increase of the repressive mark H3K27me3 creating new focal H3K27me3 domains [54, 84].

However, despite previous reports, a later study found no direct correlation between H3.3G34R chromatin incorporation and changes in H3K4me3, H3K36me3, or H3K27me3 levels when H3.3G34R was knocked out in patient-derived DHG H3.3-G34R paediatric cell lines [85]. In contrast, H3.3G34R directly impaired the recognition of H3.3K36me3 by ZMYND11 (Box 1), a specific reader of H3.3K36me3 and a transcriptional repressor of highly expressed genes. The presence of H3.3-G34R at promoters and gene bodies of master regulators of the forebrain development impaired the recruitment of ZMYND11 and thus maintained the high expression of these genes. This, consequently, amplifies the forebrain-specific transcriptional program, including key regulators like *FOXG1*, *DMRTA2* and *SOX2*, which lock cells into a proliferative, progenitor-like state associated with forebrain NSC identity [85]. In fact, loss of *FOXG1* restores senescence (via *CDKN1A/p21* upregulation) and blocks tumour initiation in patient-derived cells, underscoring its role as a regionally restricted enabler of transformation [85].

The epigenetic consequences of the less common H3.3G34V mutation are still poorly understood. While it likely also impairs SETD2 function, its inhibitory effect appears weaker than H3.3G34R [86]. In fact, mice carrying H3.3G34V/W mutation showed more modest loss of H3K36 di-methylation and tri-methylation compared to H3.3G34R [87]. In contrast, in this model, H3K27me3 was increased in G34V/W and not in G34R mice [87]. However, it remains unclear whether the prognostic differences between patients with each mutation stem from these distinct epigenetic alterations or from differences in co-occurring mutations.

H3.3G34R/V DHGs exhibit a unique DNA methylation signature [11]. Mechanistically, H3.3G34R mutation disrupts H3K36me2/3 deposition, leading to mislocalisation of DNMT3A from intergenic regions to CpG islands, resulting in widespread loss of non-CG and focal gain of CG methylation [87].

DHG H3G34mut also carry loss-of-function mutations in another epigenetic regulator, the H3.3 chaperone *ATRX* in 87.5% of cases [83]. ATRX facilitates the deposition of H3.3 in pericentromeric and telomeric heterochromatin, and a recent study found that the loss-of-function of ATRX together with H3G34R/V, mimicking the clinical presentation, in DHGs synergistically drives alternative lengthening of telomeres (ALT) [88]. In line with this evidence of the co-dependency between ATRX and H3.3G34R, studies using mouse models of DHG showed that the overexpression of H3.3G34R leads to the enrichment of neuronal markers in the tumours, the cell of origin of DHGs, only when ATRX is lost [89].

### Developmental window and cell of origin of DHG H3G34mut

The developmental window for DHG H3G34mut is postulated to align with either a prenatal window (human second trimester) or in the postnatal sub-ventricular zone [90], but has not been extensively studied [89, 91] . Regardless, studies have successfully generated DMG-HG34mut tumours postnatally, with anatomical restrictions to the ventral forebrain. Genetically modified ventral forebrain/hindbrain neural progenitor cells with a triple mutation (H3.3G34R ATRX KO and TP53KO) and the addition of N-Myc in immunodeficient mice resulted in only the ventral forebrain neural precursor cell group reaching clinical endpoint, demonstrating specificity of the ventral forebrain region for tumour induction [92].

In line with the anatomical specificity of the forebrain, ChIP-sequencing in G34R tumour samples showed elevated transcription factors specific to forebrain development [90]. The removal of G34R in tumour cells caused a significant downregulation of genes associated with forebrain development, validating that H3.3-G34R reinforces forebrain transcriptomic circuitry [85]. Also, H3.3-G34R induces a cytostatic response in the hindbrain but not in the forebrain, highlighting the importance of locoregional context specific to DMG H3G34mut [85].

The cell of origin in DHG H3G34mut is projected onto a neuronal cell of origin in contrast to H3K27M DMG (Fig. 2). The DHG H3G34mut tumours may have co-driver *PDGFRA* mutations which provide astrocytic features, hence why they are still classified as gliomas [90]. However, these tumours best reflect various stages of maturity in GABAergic interneuronal developmental states [90, 92, 93]. In keeping with the restricted interneuron cell fate, H3K27ac marks were absent at the promoter of the *OLIG2* gene which is required for oligodendrocyte cell fate, and super enhancer analysis further confirmed upregulation of intraneuronal lineage precursor markers (*DLX1,2,5,6, SOX2, CDK6*) [90]. Additionally, a Genetically Engineered Mouse Model (GEMM) expressing the H3.3G34R mutation and ATRX loss in Nestin + progenitor cells in neonatal mice generated tumours expressing early neuronal markers *Stmn2*, *Nefm* and *Nefl,* [89]. In addition, DHG H3G34mut tumours have distinct neuron-glioma interactions to DMG-H3K27M, outlining their different neuronal cell of origin [93].

Two recent single cell-RNA sequencing studies further unraveled the cellular heterogeneity of DMG-H3G34 [90, 93]. While predominantly maintaining a profile of cortical GSX2/DLX + interneuron progenitors, subpopulations of radial glial, neural progenitors and astrocytic signatures also exist. Albeit the heterogeneity, DHG-HG34 super-enhancers mainly regulate the interneuronal progenitor populations [93]. In a subset of tumours, identification of spatial structures in DHG-H3G34mut was found to resemble ‘nests’ of early migratory DCX + interneurons outlined with Nestin + progenitors [93]. Interestingly, these nest-like structures were reflective of DCX + neuroblasts found in human second trimester subventricular zone.

Taken together, cellular and transcriptomic features of DMG-H3G34 resemble a prenatal window comprising primarily DCX + interneurons outlined by Nestin + progenitor cells in the subventricular zone that may be vulnerable to oncogenic transformation.

## Posterior fossa A ependymoma (PFA-EPN)

Ependymomas (EPN) are malignant glial tumours of the central nervous system that arise from ependymal cells. In paediatric patients the majority of ependymomas occur intracranially (90%) and over 60% occur in the posterior fossa [94]. Posterior fossa ependymomas are subdivided into two major molecular subgroups: Posterior fossa A (PFA**)** and Posterior fossa B (PFB) based on their DNA methylation pattern [32]. While PFB usually arise in young adults, PFA has an earlier onset and poorer prognosis with most diagnoses occurring before 5 years old [94, 95]. Here we will focus on PFAs which have a clear epigenetic driver, including global loss of H3K27me3 and a distinct DNA methylation profile [94, 96, 97].

### Epigenetic alterations of PFA-EPN

PFA-EPN is a classic example of a paediatric brain tumour driven by epigenetic changes, since it has very few recurring genetic mutations and instead shows distinctive alterations in its epigenome [95]. Around 95% of PFA tumours aberrantly express the EZHIP protein, which mimics the H3K27M oncohistone and potently inhibits PRC2 [94], while H3K27M mutations are found in the remaining ~ 5% in a mutually exclusive manner [97]. Similar to DMG H3K27a, PFA tumours exhibit globally reduced H3K27me3 and compensatory increases in H3K27 acetylation [43, 44, 98]​.

PFA has specific epigenomic features that have been used to stratify ependymomas. In fact, H3K27ac distribution, in particular at superenhancers, is used to subclassify ependymomas subtypes [43]. Methylome analyses reveal that PFA tumours frequently display a CpG island methylator phenotype (CIMP), characterised by widespread hypermethylation across CpG sites and silencing of gene expression via promoter methylation [96]. This hypermethylation leads to transcriptional repression of genes involved in neuronal and glial differentiation. PFA’s DNA methylation landscape is quite different to DMG’s, where changes are more focal and heterogeneous [66, 67], often reflecting cell-of-origin and regional identity found in PFA [96]. A recent 3D-genome profiling study showed that EZHIP-driven chromatin reconfiguration creates strong long-range interactions between B compartment regions, named “TULIPs” [70]. These domains are marked by H3K9me3 (Box 1) and recur at predictable genomic coordinates that are similar to stem and progenitor cells. Of note, H3K27M DMG tumours did not display a TULIP-like chromatin architecture, suggesting that EZHIP’s capacity to generate TULIPs may not stem solely from PRC2 inhibition but could depend on the specific epigenetic state of the cell of origin or the developmental timing unique to PFA[70]. To confirm this model, future studies should examine the 3D chromatin structure of rare H3K27M⁺ PFA cases and EZHIP⁺ DMGs to disentangle PRC2 inhibition from the effects of developmental context.

The hypoxic environment of PFA’s developmental window during foetal hindbrain development has been shown to contribute to PFA’s aberrant epigenome by disrupting metabolites required for histone methylation in addition to EZHIP effects [98]. This specific environment may explain the dramatic Changes in DNA methylation and 3D chromatin structure that occur in PFA but not DMGs despite their common PRC2 inhibition mechanism. In conclusion, there are multiple and connected epigenetic “hits” in PFA: PRC2 inhibition, a unique DNA hypermethylated pattern and a repressive 3D chromatin structure that altogether locks cells in a primitive state and blocks normal ependymal differentiation enforcing malignant self-renewal.

### Developmental window and cell of origin of PFA-EPN

It is postulated that PFA’s first hit may occur at a prenatal development window [98–100], during early hindbrain development within the fourth ventricle and cerebellum [101]. This stage is most often hypoxic and displays enriched roof-plate stem cells and gliogenic progenitor populations (Fig. 2) [98]. Concordantly, PFA ependymomas are transcriptionally dependent on hypoxic signalling which reflects this early window of development [98–100].

The prenatal initiation of PFA has also been corroborated by cell of origin studies. The initial study in 2005 proposed that PFA arises from an early radial glial cell with an influence from specific Homeobox family transcription factor signalling for anteroposterior patterning [102]. However, almost two decades later, single cell RNAseq studies expanded this by showing intertumoural heterogeneity that demonstrated a large proportion of ependymal cells [77] and early neural precursors (PFN-NSC), while radial glial cells seem to be the origin for supratentorial ependymomas (Fig. 2) [103]. PF-NSC programs were enriched for transcriptional programs with stemness (LGR5, Wnt signalling), while PF-ependymal cells were enriched for ciliogenesis programs and were less proliferative. In line with the stemness capabilities, PF-NSC transcriptional signature correlated with PFA-EPN [103], while ependymal-like programs are most differentiated and correlate with better prognostic outcomes seen with ST-YAP1 and PF-B subgroups. RNA velocity signatures initiated in PF-NSC like cells showed differentiation towards three lineages: astro-ependymal, glial progenitor-like and a minor population of PF-neuronal precursor like type [103]. Thus, PFA-EPN subgroups may be developmentally stalled in their prenatal window enriched in undifferentiated PF-NSC which correlates to tumour aggressiveness.

**Table:** 

**Box 2. Models and Methods to study the three-event model** Epigenetics, developmental window and cell of origin are intimately associated as the prerequisite for epigenetically-driven brain cancers, so it is imperative to use the right models and techniques to address the intricates of these events, and these are summarized in Table 1. Historically, the initial approaches to interrogate epigenomic alterations were by either immunohistochemistry (IHC) or western blot where the total levels of the pertinent histone modification would be compared with either its normal counterparts or tumors lacking epigenetic mutations, the gold standard example is the DMG H3K27a subtypes where quantification of H3K27me3 through IHC is routinely done for suspected cases to confirm their molecular subtype. With the advances in epigenomic techniques, additional methods have been used to Further study the epigenomic consequences of these epigenetic alterations, including ChIP-seq for histone posttranslational modifications, ATAC-seq for chromatin accessibility, HiC for 3D chromatin remodelling [69] and DNA methylation [40]. This information has been subsequently used to interrogate the cell of origin when tumor specific enhancers were characterised using marks like H3K27ac and H3K4me1 [59, 61, 78] or tumour subtyping in the case of DNA methylation [35, 94, 104]. High throughput scRNAseq has significantly improved the level of resolution to fine-tune the cell lineages. Cell atlases based on scRNAseq in the normal developing brain have enabled the precise annotation of the cell origin when compared to scRNAseq in tumors [105]. Conversely, to validate the cell of origin and the anatomic location of brain tumors, it is essential to have adequate *in vitro* and *in vivo* models that can accurately reproduce the spatial-temporal cell lineages from which these tumors originate. Building reliable models have been challenging, where *in vitro* cell systems using NSCs from different areas of the brain have been used to transduce the epigenetic drivers, however contrasting results have been found [58, 61, 85], proving that having all of the 3 factors right is critical to successfully create these models. Different types of *in vivo* models have been generated to validate the cell of origin and oncogenic role of the epigenetic drivers; conditional genetically modified mice or mice that undergo in utero electroporation at specific stages of embryonic development have been presented in this review and summarized in Table 1. We envision that the combination of the knowledge generated from scRNAseq, scATACseq, and scHiC atlases in pediatric brain cancer and the normal brain development, with the use of NSCs from the anatomical location, are critical to build *in vitro* and *in vivo* models. The induction of the epigenetic alteration at the right developmental window in the adequate cell lineage would be the ideal way to study the three-factor model for the origin of epigenetically-driven pediatric brain cancers.	

## Conclusions and future directions

The establishment of the order and validity of the three-event model in epigenetically driven paediatric brain cancers is critical to establish adequate *in vitro* and *in vivo* models, which to date has proven to be challenging due to the still unknown order of events and/or the heterogeneity of the cell of origin for some subtypes. An informed model design based on the multiomic data from patients and mouse models where different timepoints, which include prenatal and postnatal stages, are analysed for cell types and epigenomic changes will be critical to recapitulate and fully understand the tumour formation and progression of these epigenetically driven cancers. Emerging models and technologies like organoids and lineage tracing mouse models hold great potential to improve our understanding of the three-factor model.

Understanding the main drivers of tumour development and how they extend beyond tumour transformation is critical to develop a targeted therapy towards the causes of tumour aggressiveness. To this end, additional models using patient-derived cell lines together with CRISPR screening could be the ideal strategy. This will answer crucial questions about whether the drivers of tumourigenesis are further needed for tumour maintenance, and if they are genetic dependencies. In turn, this will enable the validation towards a cell of origin-based therapy.

## Data Availability

No datasets were generated or analysed during the current study.
